# Quality of life in adults with muscular dystrophy

**DOI:** 10.1186/s12955-019-1177-y

**Published:** 2019-07-15

**Authors:** Matthew F. Jacques, Rachel C. Stockley, Gladys L. Onambele-Pearson, Neil D. Reeves, Georgina K. Stebbings, Ellen A. Dawson, Lynne Groves, Christopher I. Morse

**Affiliations:** 1 Musculoskeletal Science & Sports Medicine Research Centre, School of Healthcare Science, Faculty of Science and Engineering, Manchester Metropolitan University, Manchester, UK; 2 School of Nursing, University of Central Lancashire, Preston, UK; 3 Research Institute for Sport and Exercise Science, Liverpool John Moores University, Liverpool, UK; 4 The Neuromuscular Centre, Winsford, Cheshire UK

**Keywords:** Quality of life, Strength, Fatigue, Activities of daily living, Self-efficacy, Muscular dystrophy

## Abstract

**Background:**

Muscle weakness is a defining characteristic of Muscular Dystrophy (MD); however, yet while speculated, objective measures of muscle weakness has not been reported in relation to quality of life in adults with MD.

**Objectives:**

1) compare the self-reported QoL of adults with Duchenne MD (DMD), Beckers MD (BMD), Limb-Girdle MD (LGMD) and Fascioscapulohumeral MD (FSHD, and a non-MD (CTRL) group; 2) present and compare between groups measures of Impairment (Muscle Strength and Activities of Daily Living) and Perception (Fatigue, Pain and Self-Efficacy); and 3) identify associations between QoL domains and measures of Impairment and Perception (See above).

**Methods:**

Seventy-Five males, including MD classifications DMD, BMD, LGMD, FSHD and CTRL, completed measures for QoL, Knee-Extension Maximal Voluntary Contraction (KEMVC), Fatigue, Pain, Self-Efficacy and Activities of Daily Living (ADL).

**Results:**

QoL was lower across many domains in MD than CTRL. FSHD scored lower than DMD for mental wellbeing domains. KEMVC associated with Physical-Function domain for BMD. Pain, Self-Efficacy and ADLs associated with QoL domains, with Fatigue the most consistently associated.

**Conclusion:**

The present study identified differences between MD classifications within self-perceptions of mental-health. Muscle weakness is a defining feature of MD; however, it doesn’t define QoL in adults with MD. A greater understanding of mental wellbeing, independence, and management of fatigue and pain, are required to improve QoL for adults with MD.

## Background

Muscular Dystrophy (MD) is a group of neuromuscular conditions, characterised by progressive muscle wasting and weakness [1]. Duchenne MD (DMD), Becker MD (BMD), Limb-Girdle MD (LGMD) and Facioscapulohumeral MD (FSHD) are 4 types of MD where the impairments are uniquely comparable within the broader MD classifications, by being associated with defects in the proteins of the sarcoglycan complex [2–6]. While these MD types are comparable in impairments associated with the sarcoglycan complex, their presentation and clinical progression are specific to their own condition, and therefore each MD type should be considered independently. DMD is the most severe of the MD described here, with loss of ambulation typically by the age of 12, by comparison BMD is broadly a milder and more varied condition, with loss of ambulation typically not until adulthood [7, 8]. LGMD and FSHD are classically characterised by local weakness consistent with their names [9, 10]. Irrespective of condition, all MDs typically present with declining muscle strength and eventual loss of ambulation [11], which are likely to reduce independence, and the self-perception of physical function [12]. The loss of muscle strength and function is commonly seen as a defining feature of MD, despite however, QoL reported as typically lower in adults with MD [13–17], the impact on perceived QoL through objectively methods of muscle strength remain unreported in adults with MD [18].

QoL represents an individual’s perception of their physical, mental and social functioning [19], and is a meaningful measure of how a clinical condition may be impacting an individual. QoL has been reported previously in adults with MD, however, when multiple classifications of MD have been included within the same report, classifications have been typically pooled [13, 14, 20, 21]. Similarly, reviews of QoL in adults with MD have typically grouped MD within ‘neuromuscular disorders’ or ‘muscle disease’ groups, whereby evidence is diluted by other conditions, such as myotonic dystrophy, idiopathic inflammatory myositis and inclusion body myositis [15, 22]. The variations in mutative-genetic cause [3], and clinical progression [7–10], of each condition strongly suggest that each classification should be recognised and assessed independently, in order to detect possible differences in QoL between classifications of MD. In addition, greater understanding of the factors associated with QoL within different classifications of MD is required.

Given the progressive loss of strength and function associated with MD [23], research to date has focussed on the physical aspects of individual’s lives, such as respiratory function [24] and muscle strength [18]. Associations between QoL and objective measures of muscle strength however, remain largely unreported within adults with MD [23], with limited previous research using functional scales which lack sensitivity [14, 25, 26]. By comparison, loss of muscle strength has previously been correlated with QoL in children with DMD [18]. More broadly in MD, a loss of muscle strength limits the ability to walk and perform functional tasks [11, 27], which are both facets contributing to QoL. In addition, increased BMI has been reported previously in adults with MD [11, 28], and shown to impact not only function [29], but also effect psychological wellbeing in overweight individuals [30]. Alternatively, psychological aspects such as self-efficacy [31], an individual’s confidence in their ability to overcome problems [32], may provide a greater insight into QoL than physical impairment alone and has been shown to be positively associated with QoL in other clinical conditions [33, 34]. Other perceived measures, including fatigue and pain, are also likely to impact upon QoL in all MD groups, and have previously been shown to impact QoL in adults with DMD and FSHD [16, 25, 35], and have been proposed when discussing neuromuscular and muscle diseases more broadly [15, 22], however have not reported in adults with BMD or LGMD.

This study therefore, aimed to 1) compare the self-reported QoL of adults with DMD, BMD, LGMD and FSHD, and a non-MD control (CTRL) group; 2) present and compare between groups measures of Impairment (Muscle Strength and Activities of Daily Living) and Perception (Fatigue, Pain and Self-Efficacy); and 3) identify associations between QoL domains and measures of Impairment and Perception (See above).

## Methods

Seventy-five adult males volunteered to participate in this study (*n* = 15 DMD, *n* = 18 BMD, *n* = 12 LGMD, *n* = 14 FSHD, *n* = 16 non-MD controls (CTRL), Table 1). Participants were grouped by their dystrophic condition. All participants with MD were recruited from, and tested at, The Neuromuscular Centre (Winsford, UK). None were taking part in a structured training programme, however all were receiving once weekly or fortnightly physiotherapy treatment. Control participants, were recruited from the general population, were free from any health conditions, self-reported as being recreationally active (undertaking no more than 1 h of moderate physical activity per week), and were not undertaking any structured sport/exercise training programme. CTRL participants were tested at the local university campus, using identical methods and equipment as the MD participants (other than height and body mass, see below). None of the MD or CTRL participants reported any change in their activity levels or physiotherapy provision in the 3-months prior to inclusion in this study. Ethical approval was obtained through the Sports and Exercise Science Ethics Committee, Manchester Metropolitan University, and all participants provided informed written consent forms prior to participation. All procedures complied with the World Medical Association Declaration of Helsinki [36].

### Procedures

All participants were tested in a single testing session. The same equipment was used for all participants, with the exception of seated scales for body mass measures in non-ambulatory MD participants. All participants were assessed in a seated position to ensure consistency. Anthropometric measures were performed first, followed by a strength assessment. Questionnaires for quality of life, activities of daily living, self-efficacy, fatigue and pain were completed independently. The principal investigator was present to aid with any questions, or in some cases, to tick the desired box for participants with limited upper-limb function.

### Anthropometrics

Control participants’ mass was measured whilst standing (unshod) using digital scales (Seca model 873, Seca, Germany). MD participants were weighed in digital seated scale (6875, Detecto, Webb City, Mo, USA). Slings, shoes, splints etc. were weighed separately and subtracted from the gross weight. All participants stature was calculated as point to point (index finger, elbow, shoulder and across midline) to replicate a previously used method on non-ambulatory participants [28, 37]. A correction of 3.5% was applied to the raw data, consistent with regression data from Caucasian males in order to account for the known discrepancy between height and arm span measures [38].

Body Mass Index (BMI) was calculated using the following equation [39]:

$$ BMI\ \left(\frac{Kg}{m^2}\right)= Body\ Mass\ (Kg)\div Heigh{t}^2\left({m}^2\right) $$

Participants’ age, mass, stature, ambulatory status and BMI are presented in Table 1.

### Quality of life

All participants completed the SF-36v2 questionnaire, a reliable and validated measure, with eight domains of quality of QoL [40, 41]. The constructs for the domains of QoL are Physical Functioning, Role-Functioning Physical, Role-Functioning Emotional, Social Functioning, Bodily Pain, Mental Health, Vitality and General Health [42]. All measures are scored out of 100, with higher scores representative of better health, better function and less pain. The SF-36v2 has been used and validated extensively in the general population; however, it has also been used in dystrophic populations [13, 24, 25, 43]. In addition to the eight domains within the SF-36v2, data is also presented as Total Mental and Total Physical component scores. All data was analysed using Health Outcomes Scoring Software 4.5 (QualityMetric Health Outcomes™, Lincoln, United Kingdom).

### Impairment

#### Muscle strength

Knee extension maximal voluntary contraction (KEMVC) torque was measured using methods replicative to Quantitative Muscular Assessment, as is commonly used in dystrophic studies [27, 44], for which a full description and reliability data have been reported previously [11]. Below is an overview of the measurement of KEMVC. Despite some participants being non-ambulant, testing was designed to allow all participants to participate, of which all participants were able to produce a measurable force using this technique.

The self-reported dominant leg was measured, if participants were unable to recognise a dominant leg, the right was used. All participants were assessed in a seated position with hips and knees at 90°, with non-ambulant participants remaining in their manual or power-wheelchair. A strap with a load cell (Zemic, Eten-Leur, Netherlands) attached was fastened around the ankle and perpendicularly to a weighted support bar. The strap length was shortened until the strap was taut between the load cell and limb, while maintaining limb position. All participants were verbally encouraged to extend their leg as hard as possible throughout their maximal effort. The load cell was calibrated prior to every testing session. The load cell was connected to an analogue-digital converter and displayed in real-time on a computer screen, using a self-coded program (MyLabView, National Instruments, Berkshire, UK).

Three trials were performed per participant, with one minute breaks between trials due to the high fatigability associated with these conditions [45]. The peak KEMVC force (N) was converted to torque (N.m), with KEMVC torque used for analysis, as the product of force and lever arm, measured from the axis of rotation at the knee joint to the point of linear force (centre of strap) [46].

#### Activities of daily living

Activities of Daily Living (ADL) were assessed using the 22-item Nottingham Extended ADL Scale (NEADL). Respondents record what they had done over the last few weeks, with possible answers “Not at all”, “With help”, “On your own with difficulty”, or “On your own”. To increase sensitivity, scores were allocated using a Likert scale “0–1–2-3” [47, 48], rather than “0–0–1-1”, therefore scores ranged from 0 to 66 with higher scores representing greater independence. The NEADL has been previously validated in other clinical conditions [49, 50].

### Perceptions

#### Fatigue

The 8-item Fatigue Severity subscale of the Checklist Individual Strength (CIS) was used for analysis (hereafter referred to as CIS Severity). Each item was scored on a 7-point Likert scale where higher scores indicate a higher degree of fatigue severity. Reliability of CIS has been previously reported as good (α = .82–92) [51] and has good discriminate validity [52]. The CIS has been used previously to identify chronic fatigue in adults with FSHD [53, 54].

#### Pain

A Visual Analog Scale (Pain VAS) of pain was used to quantify the level of pain felt by participants over the 7 days preceding assessment. VAS is a common method of pain assessment [55] and used in many conditions [25, 56, 57]. Participants were given a 10 cm straight line, with at one end “No Pain”, and the other “Worst Possible Pain”, and instructed to mark where, on average, they felt their pain over the preceding 7 days was on the scale. The mark was then measured and presented as distance (cm) from the “No Pain” end.

#### Self-efficacy

The General Self Efficacy Scale (GSES) [58] was considered most suitable for our population as most Self-Efficacy scales are rehabilitative, and focus on improvements or return to physical status, and were deemed invalid for a degenerative muscle condition. In contrast, the GSES focuses on overcoming problems rather than rehabilitation. The GSES is a 10-item scale, using a 4-point Likert Scale for each question. Possible responses to questions are: *not at all true* (1), *hardly true* (2), *moderately true* (3), and *exactly true* (4), resulting in a total score between 10 and 40. High reliability, stability, and construct validity have been confirmed previously [59–61].

### Statistical analysis

All analysis was performed using IBM SPSS Statistics v21 software. The critical level of statistical significance was set at 5%. All data is presented as mean ± SD, unless stated otherwise in the table legend. Tests for parametricity were performed upon participants’ anthropometrics and KEMVC, with all questionnaire data interpreted as non-parametric. All data, except for stature was parametric. Kruskal Wallis test was used to compare between groups, with post-hoc Mann-Whitney U pairwise comparisons used where appropriate. As all groups are independent and do not interact, no bonferroni correction was used during post-hoc analysis. Stature was compared between groups using a one-way ANOVA, and Tukey’s used for post-hoc comparison. Spearman’s rank correlation analysis were used to identify associations between variables and domains of QoL with ±.30–.49 considered weak, ±.50–.69 considered moderate and ± .70+ considered strong.

## Results

### Participant characteristics

Data detailing participant characteristics is summarised in Table 1. DMD were younger than all other groups, and FSHD were older than CTRL. No differences were found between groups for stature. DMD were lighter than other MD groups, and LGMD were heavier than CTRL. No differences were found between groups for BMI.

**Table 1:** Participants Characteristics

	DMD	BMD	LGMD	FSHD	CTRL
n	15	18	12	14	16
Age (Years)	24.2 ± 6.1 ^B***,LG***,F***,C*^	42.4 ± 13.5	41.6 ± 11.7	47.1 ± 11.1 ^C*^	35.4 ± 12.7
Stature (cm)	172.0 ± 4.3	177.4 ± 6.0	179.6 ± 7.2	178.6 ± 8.1	177.5 ± 9.3
Mass (Kg)	73.1 ± 14.6 ^B*,LG**,F*^	86.5 ± 20.3	97.0 ± 18.1 ^C*^	86.0 ± 11.2	81.1 ± 18.2
Ambulant	0/15	10/18	3/12	10/14	16/16
BMI (kg/m^2^)	25.5 ± 4.1	27.3 ± 6.2	29.4 ± 26.6	26.6 ± 3.4	25.5 ± 3.7

Participants’ anthropometric characteristics

*DMD* Duchenne Muscular Dystrophy, *BMD* Beckers Muscular Dystrophy, *LGMD* Limb-Girdle Muscular Dystrophy, *FSHD* Facioscapulohumeral Muscular Dystrophy, *cm* centimetres, *Kg* Kilograms, *BMI* Body Mass Index, ^B^ denotes significant difference from BMD; ^LG^ denotes significant difference from LGMD; ^F^ denotes significant difference from FSHD; ^C^ denotes significant difference from CTRL; * denotes *P* < .05; ** denotes *P* < .01; *** denotes *P* < .001

### Quality of life

Data detailing QoL in the study sample is summarised in Table 2. For clarification lower scores are indicative of lower QoL, e.g. a lower Bodily Pain domain in MD than CTRL, reflects higher reported pain in MD than CTRL.

**Table 2:** SF-36v2 in MD classifications

	DMD	BMD	LGMD	FSHD	CTRL
Physical Function	1.3 ± 3.5 ^B**,F**,C***^	18.4 ± 18.2 ^C***^	6.3 ± 10.3^C***^	18.2 ± 12.5 ^C***^	95.9 ± 9.3
Role Physical	72.1 ± 26.1 ^F*,C**^	53.8 ± 32.5 ^C***^	59.9 ± 31.5 ^C***^	41.5 ± 26.7 ^C***^	99.2 ± 2.1
Bodily Pain	66.1 ± 16.4^C*^	58.6 ± 22.2^C**^	56.1 ± 24.4^C**^	48.1 ± 28.6^C***^	87.1 ± 15.8
General Health	55.6 ± 20.1 ^C**^	48.4 ± 19.3 ^C***^	43.3 ± 22.5 ^C***^	44.1 ± 24.2 ^C***^	81.7 ± 11.2
Vitality	63.3 ± 18.0^F***^	50.0 ± 24.7^C*^	51.6 ± 17.3	37.9 ± 16.9^C***^	72.3 ± 19.2
Social Function	84.1 ± 41.9^C*^	68.8 ± 30.4 ^C**^	76.0 ± 27.4 ^C*^	67.9 ± 29.7 ^C**^	97.7 ± 6.8
Role Emotional	80.6 ± 24.5^C*^	66.2 ± 32.6 ^C**^	86.8 ± 25.7 ^F*^	69.0 ± 24.1 ^C**^	98.4 ± 4.5
Mental Health	79.0 ± 14.9 ^F*^	74.2 ± 16.0 ^C*^	78.8 ± 14.6	65.0 ± 19.1^C**^	84.7 ± 8.5
Total Physical Score	34.3 ± 5.4 ^C***^	34.5 ± 6.7 ^C***^	30.2 ± 5.8 ^C***^	30.8 ± 6.3 ^C***^	56.8 ± 3.6
Total Mental Score	58.2 ± 10.5	51.8 ± 9.3	58.3 ± 9.7	49.8 ± 11.0	55.8 ± 3.6

SF36 outcomes. Presented as mean ± SD

*DMD* Duchenne Muscular Dystrophy, *BMD* Becker’s Muscular Dystrophy, *LGMD* Limb-Girdle Muscular Dystrophy, *FSHD* Facioscapulohumeral Muscular Dystrophy; ^B^ denotes significant difference from BMD; ^LG^ denotes significant difference from LGMD; ^F^ denotes significant difference from FSHD; ^C^ denotes significant difference from CTRL; * denotes significance <.05; ** denotes significance <.01; *** denotes significance <.001

The Physical Function and Role Physical domains were lower in all MD groups compared to CTRL. Within MD, the Physical Function domain was lower in DMD than BMD and FSHD. The Role Physical domain was higher in DMD than FSHD. The Vitality domain was lower in BMD and FSHD compared to CTRL. In addition, DMD reported a higher Vitality domain than FSHD. No other differences were found between groups for Vitality.

Within the Role Emotional domain, DMD, BMD and FSHD were lower than CTRL. The Role Emotional domain, was higher in LGMD than FSHD. No other differences were found between groups for the Role Emotional domain.

The Bodily Pain, Social function and General Health domains were lower in all MDs compared to CTRL, with no differences found between MD groups. The Total Physical domain was lower in all MDs compared to CTRL. No other differences were found between groups for the Total Physical domain. No differences were found between groups for Total Mental domain. Total SF6D domain was lower in all MDs compared to CTRL. No other differences were found between groups for Total SF6D domain.

### Impairment and perceptions

The measures of impairment and perceptions are detailed in Table 3. KEMVC was less in DMD, BMD, LGMD groups compared to CTRL. KEMVC was less in DMD than BMD, LGMD and FSHD groups. No other KEMVC differences were found between groups. ADL was lower in DMD than all other groups. Compared to CTRL, ADL was lower in BMD, LGMD and FSHD, respectively. No other differences were found between groups.

**Table 3:** Measures of Impairment and Perception

	DMD	BMD	LGMD	FSHD	CTRL
KEMVC (N.m)	12.6 ± 8.8 ^B***,LG***,F***,C***^	96.6 ± 60.0 ^C**^	98.2 ± 56.4 ^C*^	123.6 ± 78.2	164.6 ± 55.9
CIS Severity	34.4 ± 8.7 ^F*,C***^	33.2 ± 10.5 ^F**,C***^	34.4 ± 10.9 ^C***^	43.0 ± 5.3 ^C***^	14.1 ± 6.1
VAS Pain	2.5 ± 1.6 ^C**^	3.5 ± 2.5 ^C***^	3.6 ± 2.8 ^C***^	3.9 ± 2.2 ^C***^	0.4 ± 0.7
NEADL	13.9 ± 6.0 ^B***,LG*,F***,C***^	36.7 ± 14.4^C***^	29.3 ± 7.1 ^C***^	39.4 ± 14.6^C**^	63.6 ± 3.1
Self-Efficacy	31.0 ± 6.2	28.3 ± 5.9	31.0 ± 5.1	30.7 ± 7.5	34.3 ± 4.3

Measures of Impairment and Perception

*DMD* Duchenne Muscular Dystrophy, *BMD* Beckers Muscular Dystrophy, *LGMD* Limb-Girdle Muscular Dystrophy, *FSHD* Facioscapulohumeral Muscular Dystrophy, *CIS* Checklist Individual Strength, *VAS* Visual Analog Scale, *KEMVC* Knee Extension Maximal Voluntary Contraction, *N.m* Newton Metres, *NEADL* Nottingham Extended Activities of Daily Living; ^B^ denotes significant difference from BMD; ^LG^ denotes significant difference from LGMD; ^F^ denotes significant difference from FSHD; ^C^ denotes significant difference from CTRL; * denotes significance *P* < .05; ** denotes significance *P* < .01; *** denotes *P* < .001

CIS Severity was higher in all MD groups compared to CTRL. CIS Severity was higher in FSHD than DMD and BMD groups. No other differences were found between groups for CIS Severity. All MD groups reported higher levels of VAS Pain compared to CTRL participants. There were no differences in pain between MD groups. Self-Efficacy was not different between groups.

### QoL correlations

The following brief description of associations (or lack thereof), are summarised in Table 4 below.

**Table 4:** Associations of domains of SF-36v2

SF-36v2	BMI	KEMVC	NEADL	CIS Severity	VAS Pain	Self-Efficacy
Physical Function	–	.609^B**^	.595^D*^ .654^B**^ .751^F**^	–	–	–
Role Physical	–	–	–	−.769 ^D**^ -.759^F**^	–	.534^D*^
Bodily Pain	–	–	.613^D*^	–	-.715^B**^ -.694^F**^	–
General Health	–	–	.564^D*^	-.525^D*^ -.620^B**^ -.644^LG*^	-.602^B**^	–
Vitality	-.800^LG**^	–	.533^D*^	-.548^D*^ -.533^B*^ -.668^F**^	-.558^B*^	.590^D*^ .541^F*^
Social Function	-.643^LG*^	.544^B*^	–	-.594^F*^	–	–
Role Emotional	–	–	–	-.544^D*^ -.851^F***^	–	.570^D*^ .600^B**^
Mental Health	–	–	–	-.588^B*^ -.575^F*^	–	–
Total Physical Score	–	–	–	-.597^B**^	-.476^B*^ -.683^F**^	–
Total Mental Score	-.748^LG**^	–	–	-.884^F**^	–	–

Associations of SF-36v2 domains

*BMI* Body Mass Index, *KEMVC* Knee Extension Maximal Voluntary Contraction, *NEADL* Nottingham Extended Activities of Daily Living, *CIS* Checklist Individual Strength, *VAS* Visual Analog Scale; ^D^ denotes significant association in DMD; ^B^ denotes significant association in BMD; ^LG^ denotes significant association in LGMD; ^F^ denotes significant association in FSHD; − denotes no significant associations; * denotes association of <.05; ** denotes association of <.01; *** denotes association of <.001

#### BMI

The Social Function domain was moderately associated with BMI, while the Vitality domain, Total Mental Score and SF6D were all strongly associated with BMI, in LGMD. No other associations were identified with BMI.

#### Impairment

Physical Function domain was associated with ADL, moderately in DMD and BMD, and strongly in FSHD. The Physical Function and Social Function domains were also moderately associated with KEMVC in BMD. The Vitality, Bodily Pain and General Health domains were moderately associated with ADL in DMD. No other associations were identified with measures of Impairment.

#### Perception

The Role Physical domain was strongly associated with CIS Severity in DMD and FSHD. In addition, Role Physical domain was also moderately associated with Self-Efficacy in DMD. The Bodily Pain domain was associated with VAS Pain in BMD (strong) and FSHD (moderate).

The General Health domain was moderately associated with CIS Severity in DMD, BMD and LGMD, and VAS Pain in BMD.

The Vitality domain was moderately associated with CIS Severity in DMD. BMD moderately associated both CIS Severity and VAS Pain with the Vitality domain. Both Vitality and Social Function domains were moderately associated with CIS Severity in FSHD.

The Role Emotional domain was moderately associated with Self-Efficacy in DMD and BMD, respectively. In addition, Role Emotional domain was associated with CIS Severity in DMD (moderate) and FSHD (strong). The Mental Health domain was moderately associated with CIS Severity in both BMD and FSHD.

Total Physical Score was moderately associated with CIS Severity in BMD, while VAS Pain was associated with Total Physical Score in both BMD (weak) and FSHD (moderate). Total Mental Score showed strong associations with CIS Severity in FSHD.

## Discussion

The present study assesses QoL across four separate classifications of adults with MD, as well as presenting a range of factors that are associated with QoL. The findings show that adults with MD typically reported poorer QoL when compared with CTRL. Furthermore, the QoL of adults with different classifications of MD were largely comparable with the exception of physical function and mental wellbeing domains. The findings highlight that despite progressive muscle weakness being a defining characteristic of MD it is not consistently associated with QoL in adults with MD; specifically, KEMVC was associated with 2/10 domains in BMD only. By contrast, QoL domains were more frequently associated with ADLs (4/10 in DMD), pain (4/10 in BMD, 2/10 in FSHD), and self-efficacy (4/10 in DMD, 1/11 in BMD and FSHD). CIS Severity was the most consistent associate of QoL in adults with DMD (4/10 domains), BMD (4/10), LGMD (1/10) and FSHD (6/10 domains). In addition, higher BMI appeared a consistent negative associate of QoL in adults with LGMD (3/10).

The poorer QoL observed in adults with MD is consistent with other reports of MD and in other conditions with impaired physical function [13, 14, 20, 62]. In the present study however, differences between the QoL of adults with different classifications of MD in domains other than physical function were identified; this suggests grouping MD conditions [14, 21, 63], or even more broadly generalising into “muscle disease”, may lead to errors of interpretation regarding QoL [15, 22, 26]. Unsurprisingly, differences in Physical Function appeared consistent with the clinical progression of each individual condition [3]. As would be expected given the severity of the condition, DMD scored lowest for physical function [23, 64]. Despite the lower Physical Function score, DMD scored higher than FSHD across Vitality, Role Physical and Mental Health domains. It is possible that this could be attributed to better coping mechanisms within DMD, as has been suggested previously in adolescents with DMD [65], with psychosocial-adjustment to living reported previously in adolescents with DMD [66] and adults living with chronic illnesses [67]. Adults with FSHD may be less likely to have these coping mechanisms, possibly due to the later onset of the condition [68], leading to large changes in individual’s lives and possible comparisons to an individual’s pre-condition state. By comparison, DMD is a life-long condition [7], therefore acceptance of the condition and limitations may be easier or occur earlier. Interestingly though, adults with FSHD also scored lower than adults with LGMD for Role Emotional and Mental Health domains, despite both conditions having a characteristically later onset [1]. Nonetheless, these assumptions are speculative and require greater investigation beyond the scope of the present study.

Within MD, the level of physical impairment is typically seen as the defining characteristic of the condition [69], and was theorised as a key associate of QoL. KEMVC was however, only associated with Physical Function and Social Function domains in adults with BMD. Previous research has shown no association between the Physical Function domain and respiratory function in adults with DMD [24], however within ambulant children with DMD, KEMVC was shown as a good predictor of QoL determined Physical Function [18]. The Physical Function domain showed more consistent associations with ADL across conditions, which can be interpreted in two ways; firstly, that an individual’s level of independence, rather than function, influences QoL; and/or, secondly, that the physical function assessment of the SF-36v2 is an assessment of physical independence, rather than function. This is furthered by the reflection of the Physical Function scores upon ambulatory status, suggesting the Physical Function domain may be less appropriate for non-ambulant individuals [70], rather, the present authors suggest further development and validation of the SF-36 walk-wheel [71]. Furthermore, the use of a single strength measure of KEMVC, previously identified as relevant to ADLs of high intensity, are beyond the capacity of many adults with MD [72]. KEMVC may not be as relevant to QoL in adults with MD particularly those who are non-ambulatory. The associations of ADL with QoL, especially in adults with DMD, signifies that being able to undertake broader aspects of daily life (potentially using adaptive measures), rather than lower limb function (i.e. KEMVC), has a positive influence on QoL in DMD. The provision of support to empower adults with DMD to be able to undertake daily tasks should therefore be considered essential for the maintenance of their QoL.

Pain and fatigue levels are higher in MD groups than CTRL within the present study, consistent with those that have previously identified pain and fatigue in adults with FSHD and DMD [13, 16, 25]. The elevated pain and fatigue levels in BMD and LGMD adults are however, comparable to DMD and FSHD adults, suggesting that fatigue and pain may be symptomatic of MD rather than within specific conditions [73, 74]. The elevated fatigue of adults with FSHD in comparison to other MD groups as well as CTRL identifies a condition specific need for further investigation and condition specific interventions [53]. Furthermore, the consistent associations of fatigue and pain across the present MD conditions with aspects of QoL highlights their impact on both physical and mental well-being [53]. Similar associations between pain and fatigue have been identified in other clinical conditions [33, 75], as well as in adults with DMD and FSHD [16, 35]. This finding suggests that interventions that are known to reduce pain and fatigue in other clinical conditions (e.g. acupuncture [76], physiotherapy [77, 78], and physical activity, as has been applied in FSHD [54]), could improve QoL in adults with MD, however there is currently a lack of research associated with pain and fatigue management in these conditions [79, 80].

No differences between any of the present MD conditions and CTRL were observed for self-efficacy, which would appear as a positive outcome with physical manifestations of MD appearing to not influence an individual’s confidence to overcome problems. Interestingly however, self-efficacy was positively associated with QoL domains, particularly within the DMD group. The authors propose this could be attributed to the severe loss of physical function associated with DMD [23], subsequently individuals may develop higher problem solving and coping capabilities, resulting in higher Role Physical, Role Emotional and Vitality domains evidenced in the current study. Further interventions in the treatment strategy of those with DMD, and more broadly all of the MDs, should address the psychosocial issues identified in the present study, as they suggest possible improvements in the QoL of adults with MD.

Despite wide variance in the physical manifestation of these MD conditions, we show that all have lower QoL compared to CTRL, with some specific differences between MD classifications in individual domains of QoL. The largest differences in QoL between MD classifications were between the Physical Function domain, consistent with the classical definitions of these conditions [3], other differences were however, identified between classifications across domains associated with mental and psychological wellbeing. This finding suggests that grouping these forms of MD together when examining QoL [14] is not appropriate given the specific differences identified. Independently, a range of variables were associated with QoL domains, with the most frequent associations being found with activities of daily living, self-efficacy and pain; it was however, fatigue that was most consistently associated with multiple QoL domains, across all MD conditions.

### Study limitations

This study has two main limitations. First, MD participants were recruited from a specialist centre, where participants attended regular physiotherapy and condition management. This could be considered a limitation as to attend a specialist centre participants must be referenced by a clinician, and are therefore more likely to already be experiencing weakness, pain or fatigue, likely affecting their QoL, predisposing the associations identified. Furthermore, it is likely that there are shared characteristics of participants who are regular users of the same specialist centre, which, for example, may explain why no associations were identified between variables and social function, given that many are also likely to be in the same or similar social circles. The use of health and rehabilitation centres have always been a focal point for clinical and neuromuscular research, and help to overcome issues of mobility and access when recruiting adults with neuromuscular conditions [28, 81]. Furthermore, our fatigue and pain data remains comparable with that previously reported [16, 25, 82]. Secondly, this study is cross-sectional, and while able to identify issues with QoL, future research is required to address the longitudinal changes associated with progressive conditions.

## Conclusion

Differences identified in domains of QoL in the present investigation suggests a greater focus is required, and further investigation is needed into mental health and wellbeing, particularly in conditions such as FSHD. It is proposed that later onset of this condition may have a large impact on psychosocial aspects associated with QoL. Furthermore, ADLs were only associated with QoL domains, other than Physical Function, in adults with DMD, highlighting the importance of independence in this condition. In addition, consistent associations of pain and fatigue across QoL domains, across MD classifications, indicates a need for future investigation into the management and treatment of pain and fatigue within adults with MD.

## Data Availability

The datasets used and/or analysed during the current study are available from the corresponding author on reasonable request.

## Abbreviations

BIA: Bioelectrical impedance
BM: Body mass
BMD: Becker’s muscular dystrophy
BMI: Body mass index
CIS: Checklist Individual Strength
Cm: Centimetres
CTRL: Control
DMD: Duchenne muscular dystrophy
FSHD: Facioscapulohumeral muscular dystrophy
KEMVC: Knee Extension Maximal Voluntary Contraction
Kg: Kilograms
LBM: Lean body mass
LGMD: Limb-girdle muscular dystrophy
MD: Muscular Dystrophy
N·m: Newton metres
NEADL: Nottingham extended activities of daily living
Pain VAS: Pain visual analogue scale
QMT: Quantitative muscle testing
QoL: Quality of life
SD: Standard deviation
SF-36 V2: Short Form 36 Health Survey version 2
